# Nonlinear Impact of Seatback Recline Angle and Crash Pulse Magnitude on Head Injury Risk During Rear-End Impacts

**DOI:** 10.3390/s25185695

**Published:** 2025-09-12

**Authors:** Aleksander Górniak

**Affiliations:** Laboratory of Vehicle Dynamics and Safety, Department of Automotive Engineering, Mechanical Faculty, Wrocław University of Science and Technology, Na Grobli 13, 50-421 Wrocław, Poland; aleksander.gorniak@pwr.edu.pl

**Keywords:** sled test, rear impact, out of position (OOP), seatback recline angle, Hybrid III dummy, head injury criterion (HIC)

## Abstract

Out-of-position (OOP) testing is increasingly important due to the development of autonomous vehicles, innovative car seat designs, and the need to verify safety in various seating configurations. This study analyzes the impact of seatback recline angle and crash pulse magnitude on head injury risk during rear-end impacts, focusing on the Head Injury Criterion (HIC). Using a sled system and a Hybrid III 50th-percentile dummy, 12 crash scenarios were examined with crash pulses of 10 g, 15 g, and 20 g and seatback recline angles of 21°, 25°, 38°, and 55°. The results showed that increasing the seatback recline angle reduces peak head accelerations but extends their duration, which, based on the Wayne State Tolerance Curve (WSTC), may increase injury risk. The HIC increased nonlinearly with higher crash pulses, especially in upright positions. The study proposes the Pelvis-to-Headrest Transmission Effect as a newly observed dynamic mechanism affecting head and neck injury risk. Findings suggest that a more reclined posture may enhance biomechanical safety in rear-end collisions, although the effect is complex and depends on multiple factors. Video analysis and *Z*-axis acceleration data confirmed that certain reclined configurations can increase compressive forces on the cervical spine, highlighting the need for comprehensive safety assessment.

## 1. Introduction

Rear-end collisions are particularly hazardous, as they represent the leading cause of cervical spine injuries of the whiplash type [1]. A defining characteristic of this injury is its unpredictability in terms of both symptoms and long-term clinical outcomes. The cervical spine serves as a critical integration point for neural connections between the body and the brain. As a result, compressive and shear forces generated during impact can lead to increased cerebrospinal fluid pressure and may cause damage to neural structures [2,3]. The issue of whiplash injury is not limited to medical aspects. It also carries legal and social implications. A review of the clinical literature indicates that chronic symptoms are more frequently associated with psychological factors, such as pain catastrophizing or high scores on the Impact of Event Scale, rather than with the biomechanical characteristics of the collision itself [1,4,5,6].

Rear-end crash tests are conducted in accordance with strictly defined testing procedures. Permissible deviations from nominal test conditions are minimal [7,8,9,10,11], which allows injury criteria to serve as reliable points of comparison. However, in the era of autonomous vehicle development and the general pursuit of enhanced passenger comfort, it becomes justifiable to consider non-standard dummy positions and non-standard crash pulses. Variations in seatback stiffness and recline geometry have been shown to affect both the distribution of cervical loads and the trajectory of head motion during rear impacts [12,13]. These structural parameters modify the timing and nature of headrest contact, influencing peak accelerations and bending moments. In out-of-position configurations, delayed contact with the restraint system increases the load transfer to the cervical spine before effective support is provided [14]. Cervical motion analysis under low-speed sled conditions reveals that even minor deviations from nominal torso posture alter intervertebral displacements, particularly in the lower cervical segments [15]. Imaging-based assessments indicate that reclined configurations deepen thoracic kyphosis, thereby modifying initial spinal curvature and influencing the body’s kinematic response during acceleration [16]. Elevated crash pulses have been associated with shorter deceleration phases and increased rebound velocity, which contribute to higher neck extension loads and a greater likelihood of secondary contact phenomena [17]. Additional tests conducted under high-severity pulses confirm the presence of seatback-induced structural feedback transmitted toward the headrest, influencing the duration and form of head acceleration peaks [18].

Rear-end collisions are typically studied under relatively low-crash-pulse conditions, based on the assumption that such scenarios involve small relative velocity differences between vehicles traveling in the same direction [7,8,9,10]. In controlled experimental settings, the crash pulse rarely exceeds 11–12 g, in line with most regulatory or research protocols. These simulated events are intended to reflect common traffic situations involving slight speed discrepancies, such as those encountered in congested urban areas. A velocity change (Δv) in the range of 15–20 km/h has been shown to correspond with such impacts and is frequently associated with whiplash-type injuries, despite the absence of structural damage [1]. Biomechanical studies confirm that, even at these relatively low Δv values, neck extension and rebound phenomena can generate significant inertial loading in the cervical spine [17,19]. Observational data from clinical and crash databases support these findings, highlighting a high prevalence of soft tissue injury in low-speed rear-end impacts [20,21]. Recent laboratory investigations have emphasized the necessity of extending rear impact testing to acceleration levels exceeding 15 g in order to account for conditions observed in more severe collisions [18,22]. Experimental results indicate that, under such pulse magnitudes, the likelihood of cervical spine overload and early rebound motion increases significantly, particularly when seatback structural stiffness is high and energy dissipation is limited [20]. Kinematic observations further reveal that rebound velocities in excess of 6 m/s can occur shortly after impact, contributing to elevated loading in the upper torso and neck regions [23]. In such cases, rebound velocities have been recorded at 6–7 m/s, and peak extension moments (My) in the lower cervical spine have exceeded 150% of the Injury Assessment Reference Value (IARV) [20,21,24]. An analysis of 515,000 cases from the NASS-CDS/CISS databases indicates that when the change in velocity (Δv) does not exceed 4 km/h, the likelihood of structural injury to the cervical spine is negligible. This finding highlights the significant role of psychosocial factors in the persistence of post-traumatic clinical symptoms, as demonstrated by studies linking chronic outcomes more strongly to individual pain perception and psychological state than to collision biomechanics [25,26]. In response to the complexity of such injury mechanisms, hybrid research methodologies have been developed. These approaches combined sled testing with varied acceleration pulses (4–20 g) and validated HBMs incorporating realistic muscle pretension, intervertebral measurements, and localized soft tissue loading analysis [13,24,27]. Complementary studies have introduced psychosocial assessment protocols into controlled volunteer testing, enabling correlation between subjective symptom reporting and mechanical exposure [28]. This multi-domain framework facilitates the identification of biomechanical safety thresholds relevant to future occupant environments. In particular, it provides a basis for evaluating passive safety under both in-position and out-of-position configurations, especially within the context of evolving autonomous vehicle interior layouts [11,29].

From a biomechanical perspective, cervical spine injury resulting from rear impacts involves several mechanisms, including axial compression, shear forces, and the ramping effect. [11,30]. Studies have shown that microtrauma to the facet joints can occur at head acceleration levels as low as 3.5–4 g [3,15,17]. Meta-analyses indicate that the protective performance of the seat depends primarily on three factors: a backset (the distance between the head and the headrest) not exceeding 100 mm, controlled energy-absorbing deformation of the seatback, and the absence of excessive headrest stiffness. Each additional 10 mm of backset may worsen test outcomes by 20–40% [21,31,32,33].

In physical rear impact testing, the BioRID II dummy remains the primary anthropomorphic test device due to its design tailored for whiplash injury evaluation. It incorporates segmented cervical, thoracic, and lumbar spine sections shaped to reflect human anatomical curvatures, which enables high biomechanical fidelity (biofidelity) in low-speed rear-end scenarios [34]. In contrast, despite its limited biofidelity in rear impact scenarios, the Hybrid III dummy is still required under regulatory protocols such as FMVSS 202aD [10]. It is also widely used in scientific studies, particularly in experimental setups aligned with regulatory standards, although it has demonstrated limitations in replicating cervical spine kinematics and seatback interaction [22,27,35]. More recently, the THOR dummy has been adopted in studies focused on non-standard rear impact conditions. Equipped with improved soft tissue instrumentation, the THOR-50M exhibits superior capability in replicating complex flexion–extension motion and capturing head-to-headrest interaction profiles [23]. However, comparative analyses indicate that this increased sensitivity results in greater seatback deflection and higher force transmission to the head restraint system, which prompts reconsideration of injury assessment thresholds [23,27]. While BioRID remains the reference standard for cervical injury assessment, its measurement reliability declines under non-standard conditions such as elevated pulse severity or seatback recline. Measurements conducted with reclined seatbacks or elevated crash pulse levels may result in dummy damage or, at best, produce data with limited reliability.

Any change in dummy position directly alters the biomechanical response during impact and therefore directly influences the final outcomes of passive safety measurements, regardless of whether the scenario involves a frontal or rear impact. For example, a frontal sled test at 20 km/h demonstrated that increasing the seatback recline angle beyond 115° extends the head displacement trajectory to 0.56 m and increases the risk of contact with the dashboard, even when four-point seatbelts are used [13]. Moreover, even a small change in seatback recline angle during a frontal impact may result in a compressive spinal injury with a mechanism similar to that of a whiplash injury, which is traditionally associated primarily with rear-end collisions [30].

In the evaluation of non-standard rear impact tests, it is considered more relevant and technically justified to apply alternative injury criteria that are not included in standard testing protocols. In response to the increasing variability in vehicle configurations and passenger postures, the recent international literature has explored rear impact biomechanics using biomechanical indicators such as the Motion Sequence Criteria (MSC), the Inter-Vertebral Neck Injury Criterion (IV-NIC), and the Neck Displacement Criterion (NDC) [36]. The MSC consider the simultaneous exceedance of threshold values in H-point displacement (over 200 mm), torso angle (over 155°), and the occurrence of ramping. The concurrent violation of these thresholds indicates unfavorable kinematics at an early stage of movement [11,36,37,38]. The IV-NIC, in contrast, is based on local intervertebral displacements, particularly in the C6/C7–C7/T1 segments. The NDC integrates translational and rotational motion of the head relative to the T1 vertebra, which increases the sensitivity of the method to seat design variations [2,39].

Most injury assessment methods rely on the relative motion between the head and thorax, making head acceleration a key parameter in evaluating occupant response. It serves as a primary input for multiple biomechanical criteria and enables comparative analysis across impact conditions [13,30]. The evaluation of the head acceleration signal provides insight into the severity and temporal structure of the impact, including peak load transfer, rebound, and contact dynamics [26,39]. In addition to its role in general kinematic analysis, head acceleration is a core component in specific injury metrics [40,41,42].

Out-of-position (OOP) testing represents one of the central challenges in rear impact evaluation. At present, these assessments are primarily conducted in numerical environments using finite element crash simulation software. Available human body models (HBMs) such as GHBMC-D, GHBMC-S, and THUMS offer high biomechanical fidelity and are widely applied in occupant kinematics studies [12]. However, inter-model variability remains a concern, as simulation outcomes can differ significantly depending on the selected HBM, even under identical boundary conditions [43]. Furthermore, results may diverge when the same dummy model is simulated in different computational solvers, such as LS-Dyna or Pam-Crash [44,45]. An additional challenge is the absence of validation data for the Hybrid III dummy in semi-reclined postures, which limits its applicability in non-standard seatback configurations [12]. As a result, numerical findings require cross-verification with volunteer-based experiments, which are feasible only under low-severity conditions, or with post-mortem human subject (PMHS) data. However, both sources provide limited coverage for reclined or OOP configurations. While numerical simulation remains a robust comparative tool for standard crash testing, its predictive accuracy in non-standard postures is not currently supported by validated experimental references. Neither ATD nor HBM models have undergone calibration procedures specific to body dynamics in OOP conditions.

Static studies using magnetic resonance imaging (MRI) have shown that increasing the seatback recline angle from 20° to 25° deepens thoracic kyphosis by an average of 8° in individuals with a lordotic neck configuration, which significantly alters the initial conditions for simulation models [16]. Postural variations such as increasing the head-to-headrest distance to 350–550 mm, forward torso lean, or torso rotation toward the center console (radio configuration) have been associated with a rise in extension moment (My) in the lower cervical spine exceeding 150% of the Injury Assessment Reference Value (IARV) [20,22,23]. This altered loading profile correlates with an elevated risk of AIS 3+ injury [39]. Even slight head flexion in volunteers under rear impact conditions at Δv ≈ 8 km/h has been shown to increase early inertial accelerations [14]. In a sled test study involving eight 50th-percentile female participants, reducing the backset distance to 100 mm decreased the injury severity index by 22%, while the absence of head-to-headrest contact led to an increase of up to 50% [40]. Seatback recline amplifies the ramping phenomenon, characterized by upward displacement of the pelvis and torso along the seatback. Rigid belt systems integrated into the seat structure, such as ABTS (All-Belts-To-Seat) and its reinforced variant SIR (Seat-Integrated Restraint), shorten the ride-down phase [46,47], increase rebound velocity, and raise neck loading [18,32], particularly under OOP conditions [20]. In contrast, modern pocketing-style seats that incorporate a flexible frame, tall headrest, and controlled seatback deformation extend the energy absorption path without inducing excessive seatback rotation [23] while improving energy distribution through controlled deformation [22].

Considering the above, a decision was made to conduct a study based on impact simulations involving different crash pulse levels and varying seatback recline angles. The study was supplemented by model validation and physical experiments carried out using a sled system. Head acceleration and signal duration were analyzed, enabling the calculation of the Head Injury Criterion (HIC). The designed experiment (DoE) also allowed for the determination of a response surface, which made it possible to evaluate the biomechanical consequences of the analyzed crash scenarios.

## 2. Materials and Methods

The experiment was conducted at the Laboratory of Vehicle Dynamics and Safety, located within the GEO-3EM Research Complex (ENERGY, ECOLOGY, EDUCATION) at Wroclaw University of Science and Technology. Rear impact scenarios were simulated using a sled system manufactured by CROWD. A vehicle seat equipped with a compatible seatbelt set was mounted on a mobile trolley. The trolley was gradually accelerated and then brought into contact with a barrier fitted with energy absorbers selected to generate the desired crash pulse.

**Figure 1:**
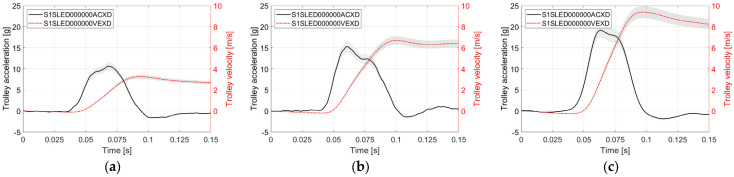
Average crash pulses: (**a**) a = 10 g, Δv = 3.5 m/s; (**b**) a = 15 g, Δv = 6.7 m/s; (**c**) a = 20 g, Δv = 9.5 m/s. The gray area represents the standard deviation (SD). Labels in parentheses follow the ISO/TS 13499 naming convention.

The experiment included three levels of crash pulse (10 g, 15 g, and 20 g) and four seatback recline angles (21°, 25°, 38°, and 55°). In rear impact testing, a low crash pulse in the range of 10–11 g is typically used to represent collisions between vehicles moving in the same direction. Therefore, the 10 g pulse was adopted as the minimum acceleration level in this study. Since biomechanics and injury criteria are closely related to the crash pulse magnitude, the range of test conditions was extended beyond standardized scenarios by increasing the pulse up to 20 g in order to gain a more comprehensive understanding of the phenomena [17,20,22,29,39]. The 15 g pulse served as an intermediate level. The crash pulses were filtered using a CFC 60 filter, in accordance with SAE J211-1. A comparison of the crash pulses is presented in Figure 1.

**Figure 2:**
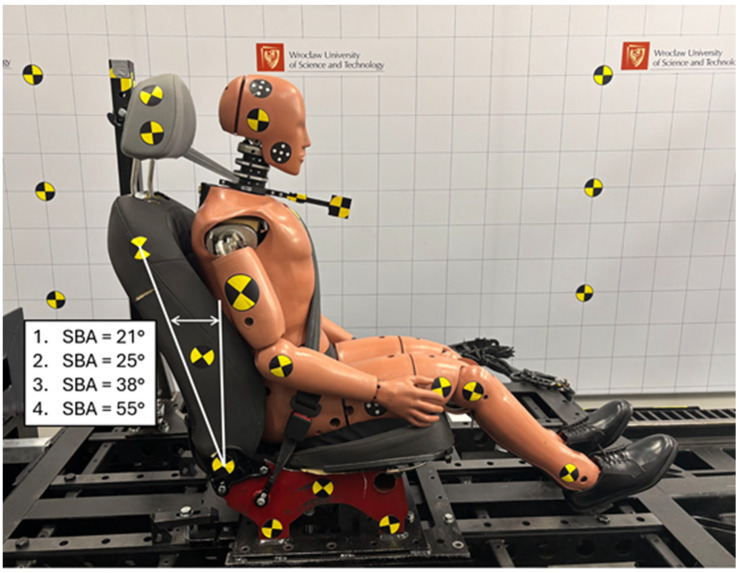
Seatback recline angles (SBAs) selected for the experiment.

The seatback recline angle (SBA) used in rear impact testing (Figure 2) is typically set either according to the original equipment manufacturer (OEM) specifications or arbitrarily, in line with relevant standards. The most commonly used nominal seatback recline angle is 21°, which was selected as one of the variables in the experiment. The arbitrarily imposed angle of 25° is specified in American standards such as IIHS, IIWPG, and FMVSS 202aD [7,8,9,10] and was therefore included in the experimental design. An angle of 38° was adopted as a mid-range value, while 55° corresponded to a fully reclined seatback configuration. The reference point was a rear impact test with a dummy seated in a vehicle seat with a 21° seatback recline angle, which corresponds to a standard seating position. A seat specifically designed and built for this study was used. The seatback was rigidly connected to the seat base, which eliminated the effect of seatback dynamic deflection. Standard production components such as foam padding and the structural frame typical of passenger vehicle seats were retained.

**Figure 3:**
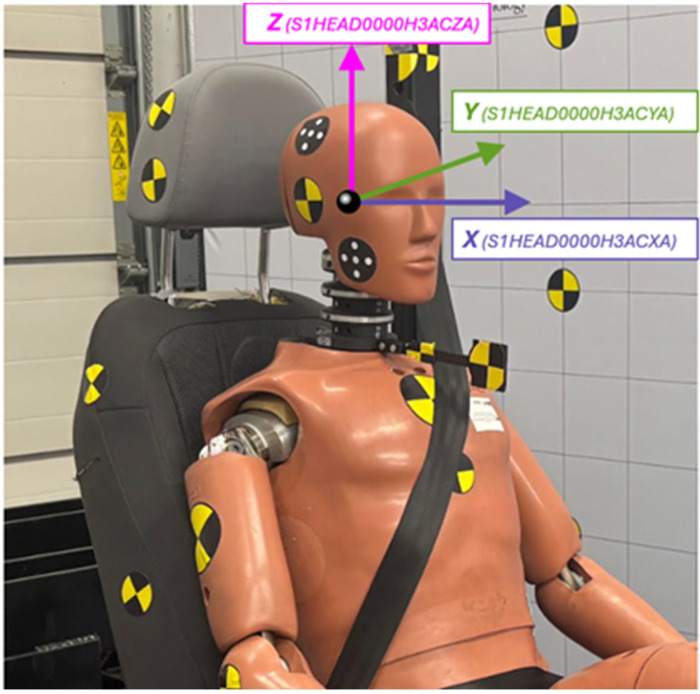
Coordinate system of the dummy’s head used in the study. Axis labels in parentheses follow the ISO/TS 13499 naming convention.

The analysis focused on head accelerations recorded at the center of gravity of the dummy’s head using a Hybrid III 50th-percentile male model. In order to assess the severity of the impact, a detailed evaluation of the acceleration signal was performed, with particular attention to the peak value and its duration. The duration of the acceleration peak directly influences the severity of the crash outcome. A shorter duration allows for higher acceleration levels to be considered tolerable for the human body [11,30,38,48,49,50]. The Head Injury Criterion (HIC) was also calculated, without arbitrarily limiting the time interval (i.e., HIC15 or HIC36 were not used). This decision was based on the fact that, in out-of-position tests, different seatback recline angles result in varying peak durations of resultant acceleration. The sensors recorded data at a sampling frequency of 15 kHz, and in accordance with SAE J211 recommendations, signals were filtered using a CFC 1000 filter. Measured data were named according to the ISO MME format (ISO/TS 13499). The adopted coordinate system is shown schematically in Figure 3. To verify the dummy’s biomechanical response based on sensor data, each test was additionally recorded using a Phantom VEO 410L high-speed camera, operating at 3000 frames per second in a maximum resolution of 800 × 1280 pixels.

The analysis of the data revealed that both head acceleration and the resulting HIC values change nonlinearly with increasing crash pulse. Therefore, an exponential regression analysis was used to quantitatively describe the relationship between the HIC value and crash pulse magnitude. For each seatback recline angle, a separate mathematical model was fitted in the form presented in Equation (1):

(1) $$HIC=a\cdot e^{bg},$$

where:

a is the regression constant,
b is the regression coefficient,
g represents the crash pulse magnitude (in g).

The use of an exponential model is justified by the mathematical nature of the HIC, which depends on both head acceleration and the duration of the head acceleration signal. The observed nonlinear increase in HIC with rising head acceleration and longer pulse duration indicates that a linear model would not be suitable for capturing the dynamics of this relationship. Traditional analysis methods such as linear regression, Student’s *t*-test, or analysis of variance (ANOVA) assume a homogenous variance distribution, which does not align with the exponential growth of the HIC observed, particularly at higher crash pulse levels. In modeling the relationship between crash pulse and the HIC, an exponential regression was selected due to its consistency with the mathematical structure of the HIC formula, which multiplies acceleration magnitude and duration in a nonlinear manner. Other nonlinear forms, such as power-law or polynomial regressions, were considered but discarded due to their lack of physiological interpretability or tendency to introduce non-monotonic behavior. Sigmoidal models like logistic or Gompertz functions were also excluded, as they impose asymptotic limits inconsistent with the continuous risk escalation observed in biomechanical experiments. The exponential model thus provides both a statistically robust and biomechanically valid representation of HIC growth across increasing crash pulses. In the exponential model, parameter a represents the baseline value (intercept), corresponding to the HIC at a theoretical crash pulse level approaching zero (g ≈ 0), where g denotes the applied crash pulse level. It reflects the inherent susceptibility of the system to crash input. Parameter b describes the rate at which the HIC increases in response to growing head acceleration. A higher b value indicates a faster and more pronounced rise in the HIC as the crash pulse increases.

The entire study was conducted in accordance with the Design of Experiments (DoE) methodology, using a full-factorial design (Table 1). A total of 12 distinct scenarios were planned, each of which was repeated three times. The entire experiment was randomized. This structure not only enabled detailed signal analysis but also allowed for the development of mathematical models in the form of response surfaces describing the influence of seatback recline angle and crash pulse magnitude on the measured head accelerations. This type of presentation makes it possible to assess general trends as well as to identify local extrema. For a more detailed interpretation, contour maps were also used. These represent the distribution of the measured variable in a two-parameter domain using color-coded isolines. Although response surfaces provide insight into the structure of the dependency, it should be noted that they represent only a single signal parameter (in this case, the peak value) without accounting for the full time history of the signal.

## 3. Results

### 3.1. Head Acceleration

An increase in crash pulse magnitude leads to a corresponding increase in head acceleration. For each of the analyzed crash scenarios (10 g, 15 g, and 20 g), progressively higher acceleration values were recorded by the sensor located in the dummy’s head. At higher crash pulse levels, the head moved more dynamically toward the headrest, resulting in stronger contact and higher head acceleration. The changes in head acceleration did not follow a linear pattern. Although the difference between successive crash scenarios was consistently 5 g, the increases in head acceleration were not uniform. This effect was particularly evident at a seatback recline angle of 21°, where a 10 g pulse produced a head acceleration of approximately 25 g. At 15 g, this value increased to about 30 g, which represents a 15.6% increase. At 20 g, head acceleration reached approximately 41 g, corresponding to a 28% increase compared to the previous scenario.

**Figure 4:**
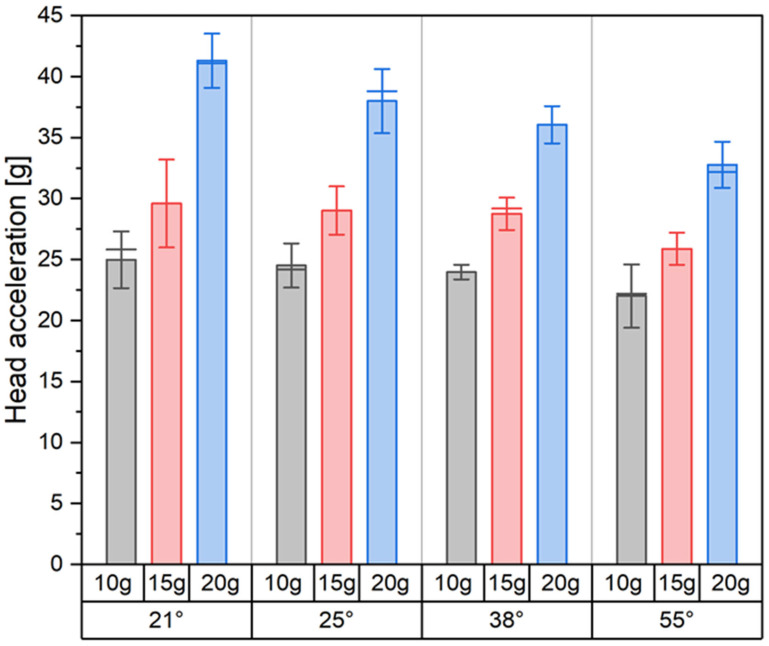
Summary of head acceleration results. Bars represent the mean value, the horizontal line indicates the median, and the vertical lines show the standard deviation (SD).

The seatback recline angle affected not only the magnitude of the recorded head accelerations but also the nature of how they developed as a function of crash pulse. A global view further shows that increasing the recline angle leads to an overall decrease in head acceleration, regardless of the pulse level. As the seatback recline angle increased, the observed nonlinearity of this relationship decreased. At a recline angle of 38°, the increases in head acceleration between consecutive crash scenarios were more uniform, which indicates a stabilizing effect of a more reclined seated posture. Even greater angles, such as 55°, further reduced the dynamic growth of acceleration. In low-energy crash scenarios, changes in seatback recline angle did not significantly affect peak head acceleration. The observed differences remained within the limits of standard deviation. For a crash pulse of 20 g, however, the influence of seatback recline angle on head acceleration became clearly noticeable. A summary of the results for all tested configurations, including different crash energy levels and seatback recline angles, is presented in Figure 4.

To fully understand the crash dynamics and potential injury mechanisms, it is essential to analyze the time history of acceleration signals, broken down by the three spatial axes (X, Y, and Z), along with their shape, duration, and onset timing. From this perspective, it is important to note that the crash pulse serves an initiating function. It acts as a trigger for the dynamic motion of the dummy, which is then captured by the sensors installed in its structure. The resulting head accelerations are a direct consequence of the motion induced by the pulse and the interaction of the body with the seat and headrest geometry. For this reason, the crash pulse is also included in the following acceleration signal plots.

#### 3.1.1. Seatback Reclined at 21°—Baseline Configuration

**Figure 5:**
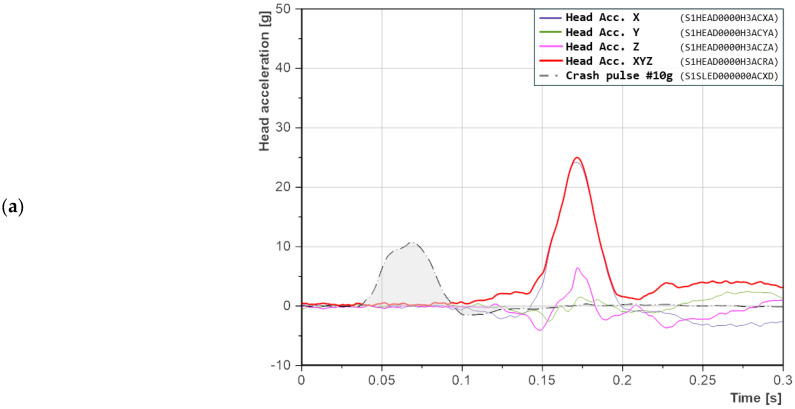

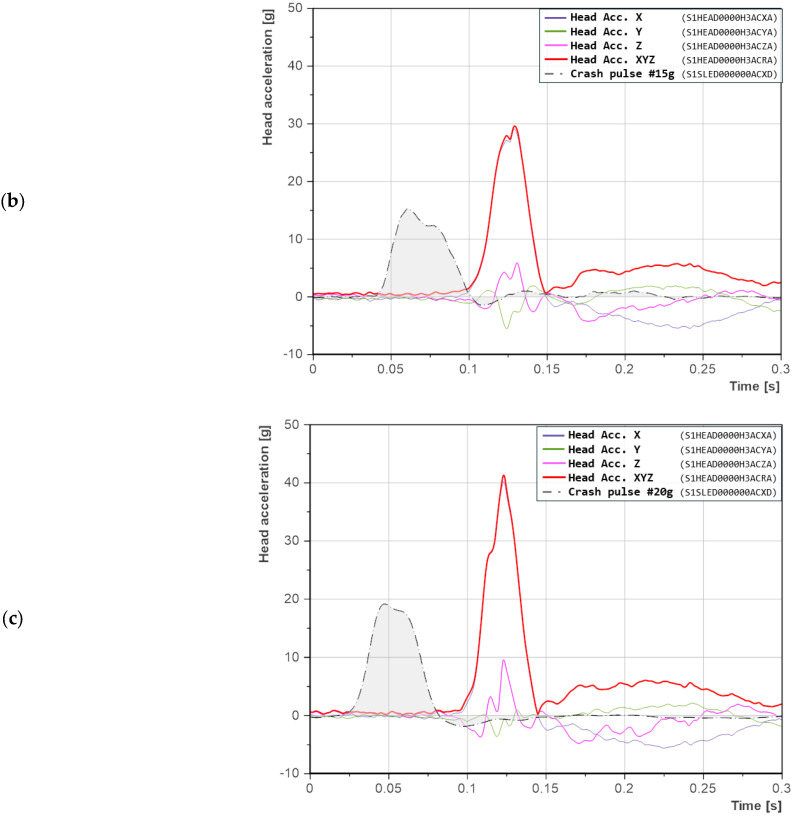
Head accelerations in the X-, Y-, and Z-axes for a seatback recline angle of 21°: (**a**) crash pulse 10 g; (**b**) crash pulse 15 g; (**c**) crash pulse 20 g. Labels in parentheses follow the ISO/TS 13499 naming convention.

The seatback reclined at 21° was adopted as the reference value, corresponding to the nominal geometry of typical car seats. The recorded head acceleration signals exhibited a regular and predictable pattern, with a single sharp peak, particularly in the *X*-axis (Figure 5). The absence of secondary oscillations and the short duration of the head acceleration suggest a simple, single-phase response: torso deceleration followed by head contact with the headrest, without the involvement of additional dynamic mechanisms.

For a crash pulse of 10 g (Figure 5a), a single distinct peak appears in the *X*-axis, clearly indicating head contact with the headrest. The *Z*-axis acceleration initially takes on a negative value, which means that the head was pulled downward. In the following phase, it increases to approximately 5 g, corresponding to neck extension during rearward motion toward the headrest. The peaks in the *X* and *Z* axes occur simultaneously. Acceleration in the *Y*-axis is negligible, indicating a relatively symmetric motion of the dummy (no lateral displacement).

With an increase in crash pulse to 15 g (Figure 5b), the acceleration peaks occur closer to the moment of impact, indicating a faster response of the dummy. Additionally, the waveforms in the X and Z axes have a similar shape, more distributed and with a local minimum. The head made contact with the headrest in a way that allowed for partial energy absorption, resulting in a peak that did not take the form of a sharp spike. The duration of the acceleration event is also shorter than in the previous case. In this scenario, accelerations in the *Y*-axis become noticeable, which may indicate asymmetric motion. The head, after rebounding from the headrest, appears to have rotated.

For the 20 g crash pulse (Figure 5c), the highest resultant head acceleration was recorded. The peak occurs even closer to the moment of impact compared to the previous case, with a difference of 6.2 ms. Here as well, the duration of the acceleration signal is shorter. The acceleration waveforms in the X and Z axes are similar in shape, with a noticeable break around 0.11 s, suggesting that the headrest absorbed part of the energy at that point. However, the subsequent acceleration peak takes on a distinctly triangular and steep profile. Interestingly, the acceleration value at the break point is comparable to that recorded in the 15 g test (Figure 5c).

#### 3.1.2. Seatback Reclined at 25°

**Figure 6:**
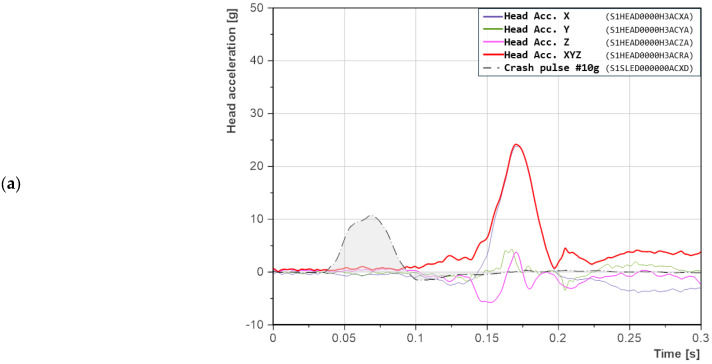

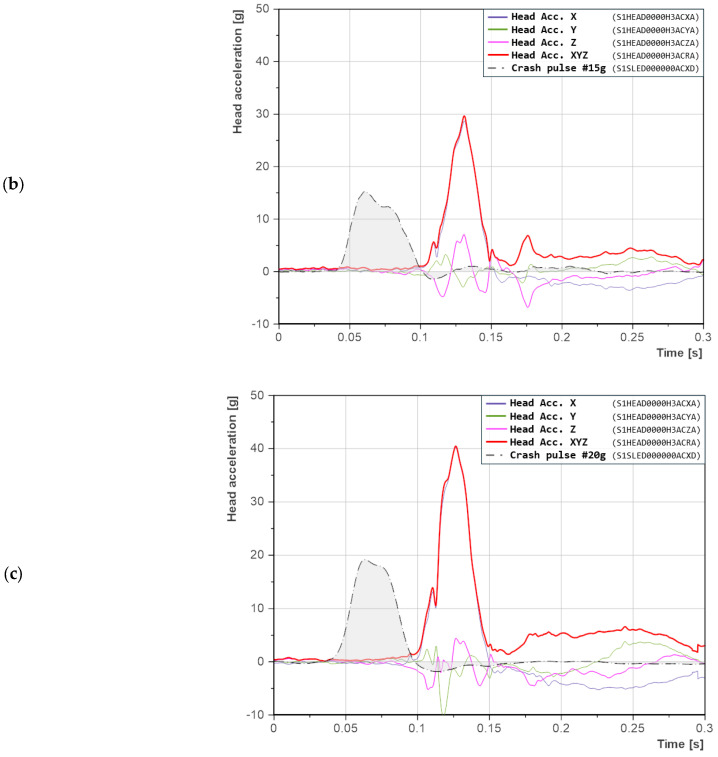
Head accelerations in the X-, Y-, and Z-axes for a seatback recline angle of 25°: (**a**) crash pulse 10 g; (**b**) crash pulse 15 g; (**c**) crash pulse 20 g. Labels in parentheses follow the ISO/TS 13499 naming convention.

Increasing the seatback recline angle to 25° resulted in a decrease in the peak head acceleration in the *X*-axis (Figure 6) by approximately 2 g on average compared to the 21° configuration. A more pronounced difference was observed in the *Z*-axis, where a wider and deeper negative signal reaching −5 g was recorded, indicating a longer path and duration of vertical head motion. This suggests that the change in angle did not affect the timing of head contact but influenced the shape and duration of the acceleration signal.

The 10 g crash pulse (Figure 6a) is characterized by a sharp acceleration peak. The duration of the resultant head acceleration remains nearly identical to the reference test, i.e., the configuration with a 21° seatback recline angle. Differences are observed in the peak value of resultant head acceleration, although they fall within the range of measurement uncertainty. In this case, a particularly pronounced negative acceleration in the *Z*-axis is evident, indicating intense downward head motion and neck extension. Based on video analysis, this downward movement was caused by the pelvis lifting off the rigid seatback, which induced torso rotation around the *Y*-axis. As a result, the shoulders and neck were pulled downward. This motion also partially reduced the resultant acceleration in the *X*-axis.

For the 15 g crash pulse (Figure 6b), a broader acceleration peak was observed in comparison with the 10 g scenario (Figure 6a). Moreover, the peak occurred closer to the moment of impact, indicating that the head made contact with the headrest more rapidly. At approximately 0.1 s, a distinct inflection in the *X*-axis acceleration curve was recorded, resulting from oscillations induced within the headrest. The impact of the pelvis against the seatback initiated a reactive motion; the headrest moved rearward and then rebounded forward. This sequence led to a dual-phase contact between the head and the headrest. Initially, the head moved rearward together with the headrest, and subsequently, it was pushed forward as the headrest returned to its original position. Acceleration in the *Z*-axis also indicated downward head movement, with a positive peak occurring simultaneously with the peak in the *X*-axis.

For the 20 g crash pulse (Figure 6c), the highest resultant head acceleration in the *X*-axis was recorded, along with the longest signal duration. The inflection in the acceleration signal, which had also been observed during the 15 g crash pulse scenario, appeared in this case with greater amplitude and a more abrupt profile. During this change in *X*-axis acceleration, the signal in the *Z*-axis stabilized, indicating that the head was in contact with the headrest and was moving rearward in a plane parallel to the ground. Significant values of acceleration in the *Y*-axis were associated with head rotation. The peak value in this axis coincided with the moment when *Z*-axis acceleration approached zero, suggesting that the head was resting against the headrest while simultaneously undergoing rotational motion.

#### 3.1.3. Seatback Reclined at 38°

**Figure 7:**
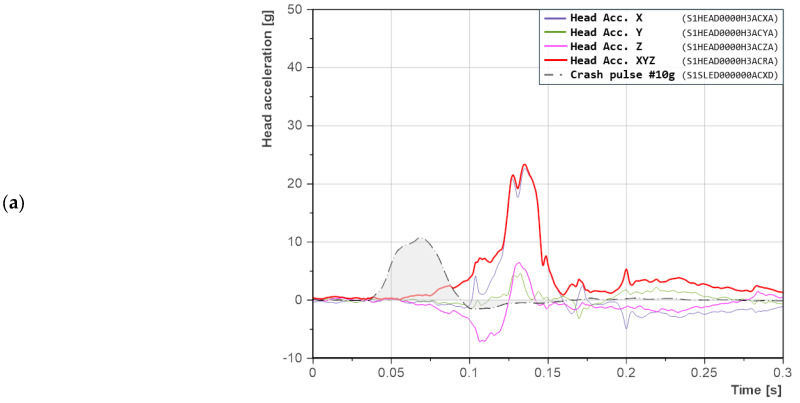

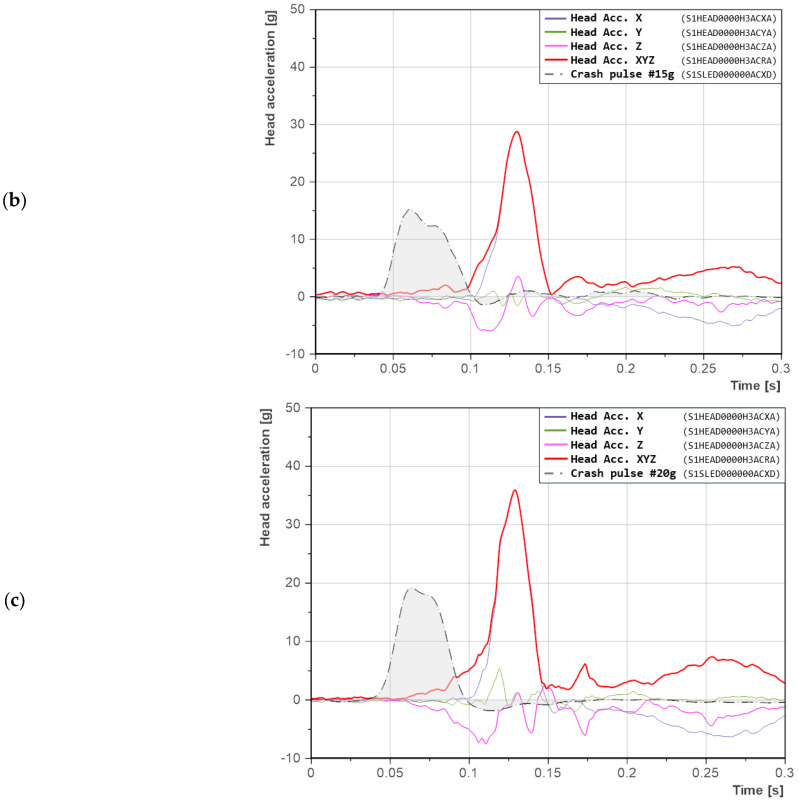
Head accelerations in the X-, Y-, and Z-axes for a seatback recline angle of 38°: (**a**) crash pulse 10 g; (**b**) crash pulse 15 g; (**c**) crash pulse 20 g. Labels in parentheses follow the ISO/TS 13499 naming convention.

In the crash scenario with the seatback reclined to 38°, all acceleration signals occurred earlier relative to the crash pulse (Figure 7). Compared to previous cases, for the lowest crash pulse level (10 g), head acceleration peaks were recorded significantly later—typically around 170 ms. Additionally, a pronounced negative acceleration in the *Z*-axis indicated the presence of compressive forces within the cervical spine during the initial phase of motion. Negative values in this axis became dominant, exceeding the positive ones, which suggested limited or no rebound of the head from the headrest.

The duration of the resultant acceleration peaks remained virtually unchanged. In the case of the 10 g crash pulse (Figure 7a), the head’s resultant acceleration peak did not exhibit a sharp profile and featured two local maxima, indicating energy dispersion during impact. Video analysis revealed that energy was dissipated due to head rotation relative to the headrest, which was further confirmed by the negative acceleration observed in the *Z*-axis. At the moment of contact with the headrest, the torso moved upward along the reclined seatback, while the head, resting against the headrest, extended rearward. This motion increased the bending moment in the upper cervical spine of the dummy. Ultimately, the head rebounded from the headrest, which manifested as synchronized acceleration peaks in both the X- and Z-axes. Additional peaks were also recorded in the *Y*-axis, indicating simultaneous head rotation. Overall, the relatively low crash pulse caused the pelvis to rebound from the seatback and slide upward along the seat surface, resulting in upward displacement of the dummy.

Increasing the crash pulse to 15 g (Figure 7b) resulted in the head’s resultant acceleration peak occurring at nearly the same time as in the previous case, with differences of only a few milliseconds. This time, however, the peak was sharp, indicating head contact with the headrest with reduced energy dissipation. Negative acceleration in the *Z*-axis again indicated the presence of compressive forces in the cervical spine, and the synchronization of peaks in the *X-* and *Z*-axes confirmed direct contact. The positive acceleration value in the *Z*-axis was considerably smaller than the negative one, which suggested that the head was primarily pressed against the headrest. This phenomenon resulted from the pelvis rebounding off the lower part of the seatback, which caused the torso to rotate around the *Y*-axis.

This behavior became even more pronounced under the highest crash pulse of 20 g (Figure 7c). In this case, positive acceleration in the *Z*-axis was nearly absent, indicating the predominance of compressive forces. Pelvic rebound from the seatback caused torso rotation, which pressed the head against the headrest. As a result, acceleration in the *X*-axis exhibited a sharp profile and short duration. It was also observed that in all scenarios, stronger negative acceleration in the *Z*-axis corresponded with increased head activity in the *Y*-axis, which indicated greater lateral rotational motion.

**Figure 8:**
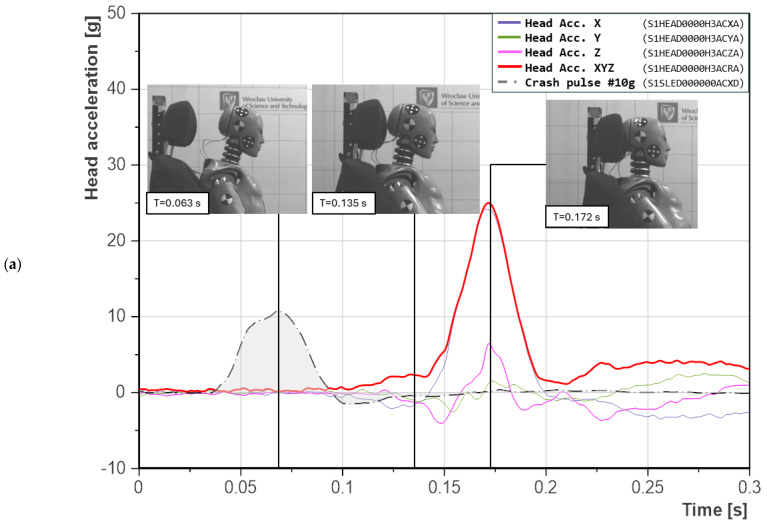

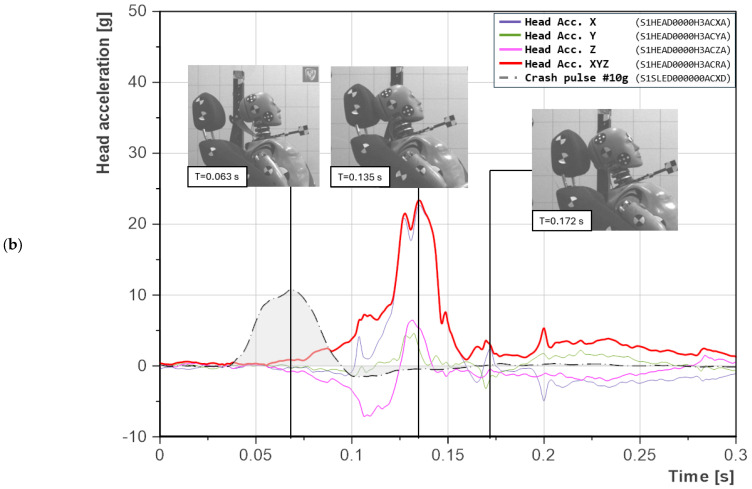
Comparison of the timing of the 10 g crash pulse peak and head acceleration peak: (**a**) seatback recline angle of 21°; (**b**) seatback recline angle of 38°. Labels in parentheses follow the ISO/TS 13499 naming convention.

The configuration with a seatback reclined to 38° exhibited a more complex head motion dynamic. A comparison of head acceleration for the 10 g scenario at seatback recline angles of 21° and 38° is presented in Figure 8. The peak head acceleration was recorded at approximately 0.135 s, while in other cases, it occurred around 0.170 s. The peak of the crash pulse itself was recorded at 0.063 s. At that instance, in both analyzed scenarios, no contact between the head and headrest had yet occurred. Another key time point is 0.135 s. For the 21° seatback configuration (Figure 8a), head-to-headrest contact had still not occurred. In the case of the 38° reclined seatback (Figure 8b), this instance corresponded to the maximum head acceleration value. In this scenario, head motion along the *Z*-axis was detected almost immediately after the peak of the crash pulse. The recorded acceleration in this axis was negative, indicating downward movement of the dummy’s head and the presence of tensile forces acting on the cervical spine. This phenomenon resulted from pelvic rebound from the seat, which, in turn, pulled the shoulders downward and caused torso rotation around the *Y*-axis. This mechanism led to earlier head contact with the headrest and earlier occurrence of maximum acceleration. In the scenario with a 21° seatback (Figure 8a), head-to-headrest contact was recorded only after 0.172 s. At the same time, in the reclined configuration (Figure 8b), the dummy had already reached its final position and remained stationary.

Moreover, in the 38° seatback recline scenario (Figure 8b), two distinct peaks in head acceleration were recorded in the *X*-axis. The first corresponded to the initial contact with the headrest, while the second resulted from a secondary force. Time-based analysis and video recordings made it possible to identify a previously undocumented mechanism, referred to as the Pelvis-to-Headrest Transmission Effect. This effect occurs when the dummy’s pelvis impacts the rigid seatback, inducing structural vibrations that propagate with a slight delay to the headrest, causing secondary head acceleration. It can be considered a new and separate dynamic mechanism relevant for the analysis of neck injuries in rear-end collisions. The signal in the *Z*-axis appeared already during the crash pulse, which indicates that early upward displacement of the pelvis led to head movement before the peak acceleration of the sled was reached. Video analysis did not reveal classical ramping, defined as upward sliding along the seatback, but instead showed torso rotation around the *Y*-axis, caused on one side by pelvic lift and on the other by downward pressure on the shoulders. Initial contact between the head and the headrest occurred at 0.127 s, marking the onset of the first acceleration peak. A short decline in acceleration followed, suggesting that the headrest yielded or translated rearward under load. Subsequently, the second and higher acceleration peak was recorded at 0.135 s, coinciding with a rapid reversal in headrest motion and recontact with the head. The plateau-like shape between the two peaks indicates that the headrest moved rearward in unison with the head before being pushed forward again. This dynamic response was initiated by pelvic impact against the lower seatback structure, which induced upward and rotational torso motion, ultimately generating delayed vibrational input to the headrest and resulting in secondary head acceleration.

#### 3.1.4. Seatback Reclined at 55°

**Figure 9:**
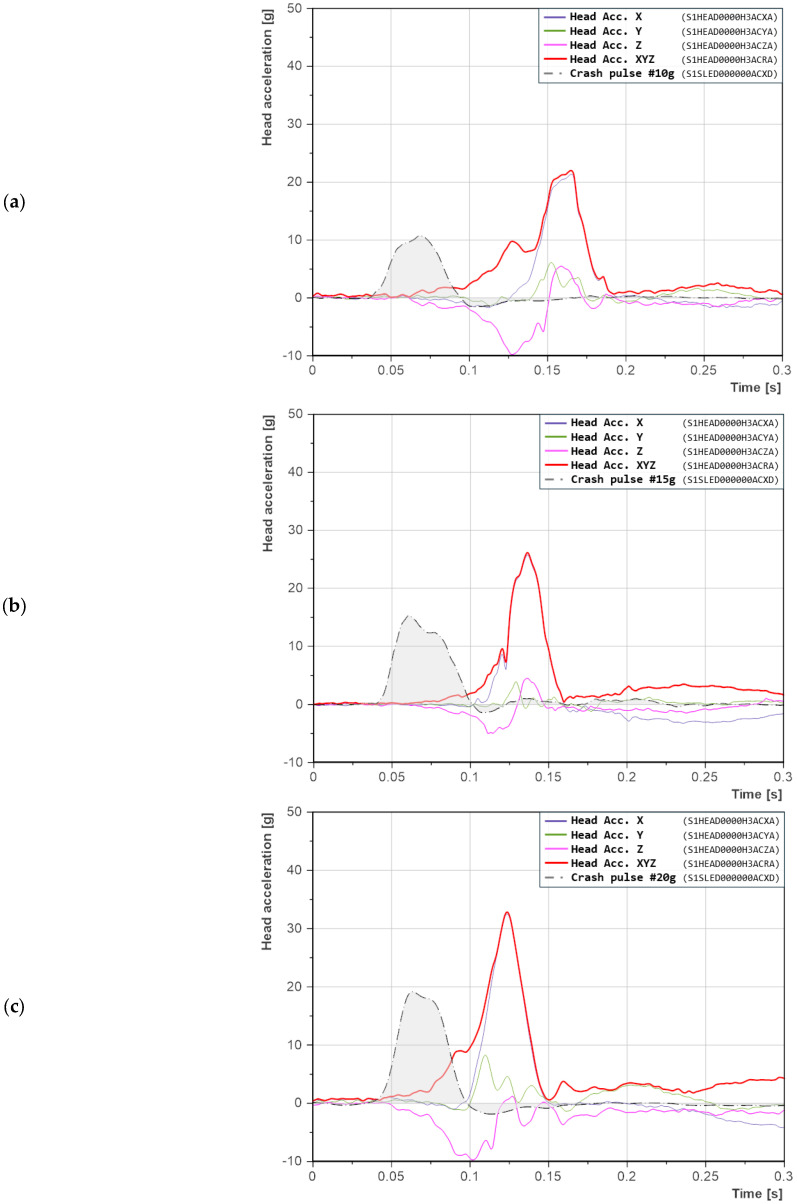
Head accelerations in the X-, Y-, and Z-axes for a seatback recline angle of 55°: (**a**) crash pulse 10 g; (**b**) crash pulse 15 g; (**c**) crash pulse 20 g. Labels in parentheses follow the ISO/TS 13499 naming convention.

In the 55° seatback configuration, the longest duration of head acceleration peaks was observed, particularly in the *Z*-axis. The recorded accelerations exhibited lower peak values compared to other cases and were significantly more extended in time (Figure 9). Moreover, the greater the crash pulse energy, the lower the value of positive acceleration in the *Z*-axis. In fact, the acceleration in this axis remained exclusively negative, indicating that the head was continuously displaced downward. Pelvic lift effectively limited upward head motion, attenuating its displacement relative to the headrest. When comparing all acceleration peaks, the longest duration of resultant head acceleration was noted, which resulted from the early onset of the *Z*-axis signal.

For the 10 g crash pulse (Figure 9a), the longest duration of resultant head acceleration was recorded. This was associated with a broad signal in the *Z*-axis, which began nearly at the time of the peak acceleration of the crash pulse itself. This indicates that the dummy’s torso started moving upward from the very beginning of the test. In this case, *Z*-axis acceleration reached nearly −10 g, a value close to that of the crash pulse. The *X*-axis acceleration, corresponding to head contact with the headrest, did not exhibit a sharp profile, which again suggested energy dissipation during impact. This phenomenon resulted from the upward sliding and rotational motion of the head along the surface of the headrest, involving lateral rotation around the *Y*-axis. Synchronized acceleration peaks in the X- and Z-axes confirmed contact between the head and the headrest. For the 15 g crash pulse (Figure 9b), an increase in head acceleration in the *X*-axis and a noticeable decrease in *Z*-axis acceleration were observed compared to the 10 g case. Additionally, at approximately 0.12 s, an inflection in the signal was recorded, indicating a complex interaction between the head and the headrest. The long duration and the steepest peak in resultant head acceleration were recorded for the highest crash pulse, 20 g (Figure 9c). The absence of positive acceleration in this axis indicated that the head was continuously pressed against the headrest. This suggests that, in this test, the dummy’s pelvis was displaced upward with the greatest intensity.

#### 3.1.5. Relationship Between Crash Pulse, Seatback Recline Angle, and Injury Risk

**Figure 10:**
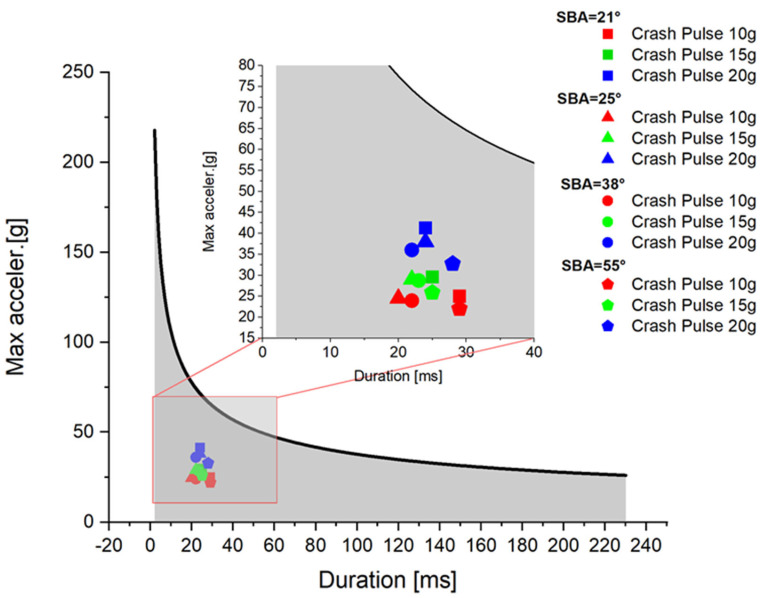
Wayne State Tolerance Curve (WSTC) with superimposed acceleration values and durations for all test conditions.

Comparative analysis across all configurations revealed two main relationships. As the crash pulse increased, peak head acceleration values also increased, most noticeably in the X- and Y-axes. However, the duration of the resultant acceleration peak did not follow a simple linear trend with increasing seatback recline angle. The shortest durations were observed at 25° and 38°, while the highest values appeared at 21° and again at 55° This divergence can be explained by the increasing contribution of the *Z*-axis signal, particularly at the 55° configuration. Since the resultant acceleration accounts for all three spatial components, the prolonged signal in the *Z*-axis—characteristic for this configuration—leads to an extended resultant duration. Biomechanically, longer force application times are relevant because, according to the Wayne State Tolerance Curve (WSTC), injury risk increases with duration (Figure 10). Nevertheless, in all cases, both the peak values and the duration of the head accelerations remained below injury thresholds, indicating no direct risk of head injury even in the most extreme test conditions.

### 3.2. Head Injury Criterion (HIC)

**Figure 11:**
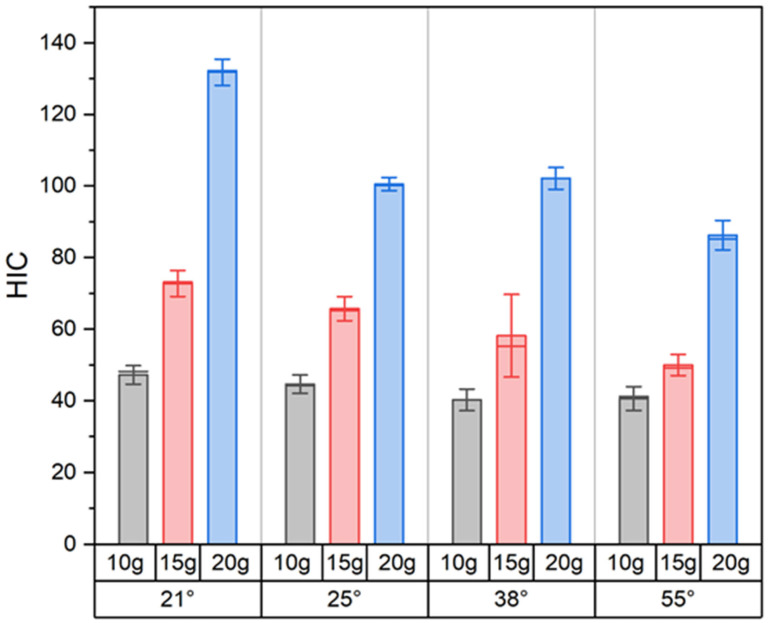
Summary of results for the Head Injury Criterion (HIC). Bars represent the mean value, the horizontal line indicates the median, and the vertical lines show the standard deviation (SD).

A comparison of HIC values for the same crash pulse magnitude across different seatback recline angles revealed minimal differences at the lowest level (10 g), with more noticeable variations observed at 15 g and 20 g (Figure 11). For the 10 g crash pulse, HIC values remained nearly constant regardless of recline angle, ranging from 41 to 47. This indicates that under low acceleration conditions, changes in seatback recline angle do not significantly affect the potential severity of head injury. The lowest HIC value in the entire test set was recorded for this configuration—10 g and 55°, with HIC = 41. For the 15 g crash pulse, a clear decreasing trend in HIC values was observed with increasing seatback recline angle. The HIC value was 72 at 21°, 61 at 25°, 58 at 38°, and 50 at 55°, corresponding to a 31% reduction between the most upright and the most reclined position. A similar, even more pronounced trend was noted for the 20 g crash pulse, where the HIC decreased from 132 at 21° to 89 at 55°, representing a 33% reduction.

The HIC values demonstrated a nonlinear increase with rising crash pulse magnitude (Table 2). For all seatback recline angles, the increase in the HIC between crash pulse levels of 15 g and 20 g was substantially greater than that observed between 10 g and 15 g. At a 38° seatback recline angle, the HIC increased from 42 to 58 (+38%) when moving from a crash pulse of 10 g to 15 g and from 58 to 103 (+78%) when increasing the crash pulse from 15 g to 20 g. The most nonlinear increase was recorded at 21°, where the HIC rose from 72 (at a crash pulse of 15 g) to 132 (at 20 g), representing an 83% increase. In contrast, the smallest increase at a crash pulse 15 g and 20 g was noted at 25°, where the HIC rose by only 51%, from 61 to 92.

Among all tested configurations, the safest setup was the one with a 55° seatback recline angle and a 10 g crash pulse (HIC = 41), whereas the highest HIC value was recorded at 21° and a 20 g crash pulse (HIC = 132). Although none of the measured values exceeded critical thresholds defined by institutions such as NHTSA or IIHS (typically in the range of 700–1000), differences between configurations reaching 30–40% are significant when assessing the relative level of injury risk.

#### Statistical Analysis: Exponential Regression

To evaluate the relationship between HIC values and crash pulse magnitude, an exponential regression analysis was conducted. The model assumed a functional form in which the HIC increases exponentially with the magnitude of the crash pulse, using two fitted coefficients as defined in Equation (1). The model fit parameter R^2^ indicates how well the exponential regression reflects the actual experimental data. In all cases, very high R^2^ values exceeding 0.95 were obtained, indicating excellent agreement between the data and the model. The best fit was achieved at a 25° seatback recline angle (R^2^ = 0.9999), while the weakest, yet still very good, fit was observed at a 55° seatback recline angle, where R^2^ = 0.95. Table 3 presents the estimated coefficients a, b, and R^2^ for each tested seatback recline angle. This summary allows for direct comparison of the HIC growth rate and the quality of the exponential fit across different seating configurations.

The analysis showed the following:

- The highest coefficient b = 0.1095 was observed at a seatback recline angle of 21°, indicating the steepest rate of HIC increase with rising crash pulse magnitude;
- The lowest coefficient b = 0.0811 was found at a 25° seatback recline angle, representing the slowest rate of HIC growth in response to changes in sled acceleration;
- The best model fit (R^2^ = 0.9999) also occurred at the 25° angle, further confirming the stability of this configuration;
- Even in the most extreme configuration (55° seatback recline angle), the model fit remained very good (R^2^ = 0.95).

The regression results clearly confirm a strong exponential relationship between HIC values and crash pulse magnitude. Regardless of the seatback recline angle, increases in head acceleration and pulse duration lead to a sharp rise in the HIC, particularly in the upper range of crash pulse values.

**Figure 12:**
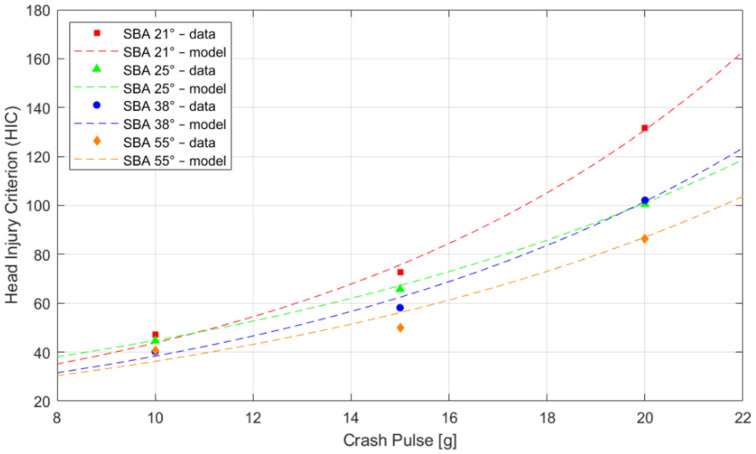
Exponential curve fitting to experimental Head Injury Criterion (HIC) data as a function of crash pulse magnitude for all seatback recline angles (SBAs).

The fit of exponential curves to the experimental HIC data as a function of crash pulse magnitude is illustrated in Figure 12. Colored points represent measured values, while dashed lines correspond to the fitted exponential functions. The steeper the curve, the greater the rate of HIC increase with rising crash pulse acceleration. The red curve, corresponding to the seatback set at 21°, is clearly the steepest and indicates the most rapid increase in the HIC. This configuration is associated with the highest injury risk. In contrast, the orange curve, representing the 55° seatback recline angle, has the flattest profile. The HIC increases much more gradually in this case, reflecting lower sensitivity to increasing crash pulses at higher recline angles. All curves exhibit a distinct upward curvature, which confirms the exponential nature of the relationship between the HIC and crash pulse magnitude. Moreover, the fitted curves closely follow the experimental data points, indicating a high degree of agreement between the mathematical model and the measurements.

### 3.3. Response Surfaces

**Figure 13:**
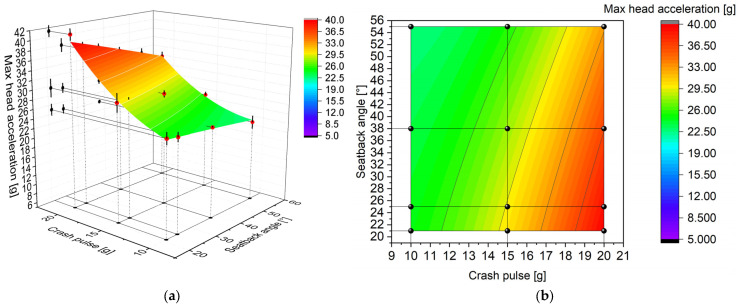
Relationship between peak resultant head acceleration, crash pulse, and seatback recline angle: (**a**) response surface with experimental data (red dots) and standard deviation (vertical lines); (**b**) contour plot with experimental data points (black dots).

The response surface and corresponding contour plot for peak resultant head acceleration are presented in Figure 13. Both the surface and the contour confirm the nonlinear relationship between the peak value and the analyzed variables. Notably, within the crash pulse range from 10 g to approximately 12 g, a dominant green color band is observed, corresponding to acceleration values between 22.5 and 26 g. As the seatback recline angle increases (*Y*-axis of the contour—Figure 13b), this range expands, indicating that a more reclined seating posture is associated with lower peak resultant head acceleration. In contrast, at lower recline angles and higher crash pulse values, acceleration increases rapidly, with the contour shifting toward color zones associated with injury-relevant levels.

**Figure 14:**
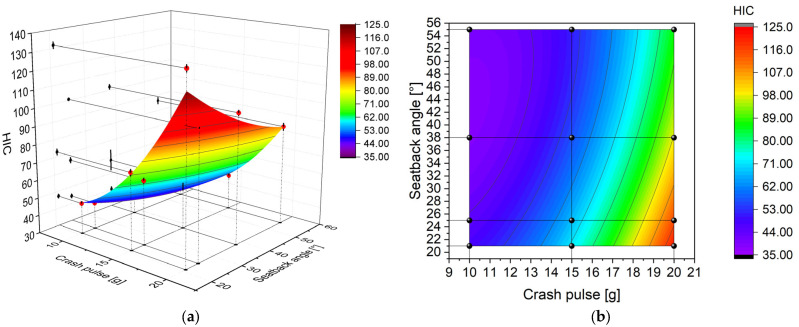
Relationship between Head Injury Criterion (HIC), crash pulse, and seatback recline angle: (**a**) response surface with experimental data (red dots) and standard deviation (vertical lines); (**b**) contour plot with experimental data points (black dots).

The response surface of the HIC plotted against crash pulse magnitude and seatback recline angle (Figure 14) shows that the highest HIC values occur only at pulses exceeding 17 g and at configurations with low seatback recline angles. This spatial distribution suggests that only strong collisions in an upright seating position produce a rapid and short acceleration pulse in the head, which corresponds to a high HIC value. A local minimum is observed at a seatback recline angle of 46° and a crash pulse of 10 g, where the acceleration signal is not only lower but also extended in time, resulting in a low HIC level. Conversely, the maximum corresponds to the configuration with the highest pulse and the smallest recline angle. In this region, the pulse is both intense and brief, accumulating into a high HIC value.

Comparing the HIC contour (Figure 14b) with the contour of peak head acceleration (Figure 13b) reveals significant discrepancies. Although both exhibit a similar overall increasing trend, with values rising alongside increasing crash pulse and decreasing seatback recline angle, their spatial distributions differ. The largest differences occur in the lower right corner of the map, corresponding to high crash pulses and small seatback recline angles. Notably, the HIC increases sharply in this region despite a more gradual rise in peak head acceleration.

## 4. Discussion

The development of autonomous vehicles and changes in their interior design, especially regarding seat construction and occupant positioning, require revising existing passive safety criteria. Standard test protocols based on upright seating positions may not reflect real conditions in new collision scenarios. Therefore, studies incorporating non-standard seatback recline angles and varied crash pulses are necessary.

The correctness of the test results was benchmarked against data from the NHTSA research database, specifically by identifying similar impact scenarios involving the use of the Hybrid III dummy. In general, the only directly comparable aspect is the nominal seat position, since the database includes reports that do not investigate reclined seat configurations. Naturally, depending on the test environment, the outcomes may vary. However, in a test conducted according to the FMVSS 202aD protocol, as reported by [51], a 10 g crash pulse resulted in a head acceleration of approximately 25 g in the X-direction, which is closely aligned with the values obtained in the present study. Although the study in [51] was primarily focused on evaluating an active head restraint system, it was noted that the activation of the head restraint did not significantly influence the measured head acceleration. Further research using a rigidly fixed seatback with a standard head restraint [52], performed under the same 10 g crash pulse conditions, also reported a head acceleration of 25 g. Notably, both the peak value and the shape of the acceleration curve in that study closely resemble the results obtained in the present research. The response of the seat to the activation of the head restraint system was also observed in [53], although in that case, the recorded head acceleration was lower, most likely due to a higher dynamic opening of the seatback.

When comparing the data obtained in the present experiment with the results presented in [54], it can be observed that, under a similar collision impulse, the values are to some extent convergent. Only the nominal seat position was used for the comparison. In the study described in [54], one of the test configurations involved the use of a standard Hybrid III dummy. In this case, a head acceleration of approximately 10 g was recorded at a collision speed of 10 km/h, while at 16 km/h, this value increased to around 20 g.

In contrast, in the tests conducted in the present study, with an impulse corresponding to 10 g and 4 m/s (i.e., approximately 14.4 km/h), the head acceleration in the *X*-axis reached a level of about 25 g. The observed differences between the results may stem from the fact that a specially designed seat was used in the present study, which eliminated one degree of freedom and completely reduced the effect of seatback deflection. Additional discrepancies at higher collision impulses may also be related to significant seatback deformation observed in the experiments described in [54]. It should be noted that [54] does not provide an acceleration curve but only reports the change in velocity (Δv), which makes direct comparison of the test conditions difficult and prevents a definitive conclusion as to whether the collisions were energetically equivalent.

The results of the conducted sled tests demonstrated a significant influence of the seatback recline angle on the dynamics of head motion during rear impacts. Two effects were particularly noticeable: a delay in the timing of peak head acceleration and an exponential increase in the HIC value. These phenomena were most pronounced in semi-reclined configurations (angles of 38° and 55°), where a desynchronization between head motion and headrest occurred. Similar dependencies were observed by Frej [13], who indicated that seatback recline angles exceeding 115° increase head rotation and the risk of cervical injuries. Although his study did not analyze HIC values, the impact of seatback geometry on head trajectory aligns with the trends observed in the present experiment. Keifer et al. [14] demonstrated that the out-of-position (OOP) posture results in delayed head contact with the headrest and increased loads occurring prior to this contact. Similar conclusions were drawn by Zehr et al. [46], who reported that a significant portion of reaction forces in rear impacts originates from the pelvis area and is transmitted through the lumbar spine to the upper body segments. These findings support the hypothesis that the observed acceleration pulse in the *Z*-axis may result from internal force transmission rather than solely from direct head motion.

The analysis of the head acceleration signal revealed two distinct peaks. The first corresponded to the initial head contact with the headrest, while the second, occurring with a delay, resulted from a secondary dynamic response. Based on temporal analysis and video recordings, a novel previously undescribed mechanism was identified called the Pelvis-to-Headrest Transmission Effect. This phenomenon involves the dummy’s pelvis impacting the seatback generating structural vibrations that propagate to the headrest after several milliseconds, causing secondary head acceleration. This effect does not arise from body sliding or classical ramping but represents a dynamic response of the seat structure. Although not previously described in the literature, a biomechanical rationale for this mechanism can be found both in reference [46] and in the MSC concept reference [37], which emphasizes the importance of pelvis stabilization. This mechanism can significantly affect injury criteria measurements, especially in semi-reclined positions, and should be considered in the design of protective systems for future vehicles.

One of the key mechanisms revealed in the study was an effect distinct from the classically defined ramping. According to the biomechanical literature, ramping refers to the upward displacement of the pelvis and torso along the inclined surface of the seatback [6,18,22,23,46]. Initially, it was assumed that a similar phenomenon would occur in the analyzed tests. However, under the conditions of this experiment, especially at large seatback recline angles and high crash pulse energy, a different mechanism was observed: instead of the entire torso sliding upward, the seatback recline caused a dynamic pelvis rebound against the seatback and lowering of the shoulder girdle, resulting in torso rotation around the *Y*-axis. This effect directly influenced the bending of the dummy’s cervical spine and the characteristics of the head acceleration signal, including a delayed peak timing. Additionally, according to the Motion Sequence Criteria (MSC) concept described by Viano [37], limiting ramping and stabilizing the hip point (H-point) are crucial for maintaining a favorable occupant motion trajectory. In the present study, it was found that in semi-reclined configurations (≥38°), the H-point shifted upward, resulting in increased HIC values, fully consistent with the MSC assumptions. Previous ramping studies mainly involved seats that allowed for deformation or rotation of the backrest, whereas in this experiment, a rigid seat structure fixed relative to the base was used. This suggests that the ramping effect is strongly dependent on the seat structure, particularly its compliance and energy absorption capacity. Kinematic analyses also highlighted that ramping is strongly influenced by the stiffness of the seatback construction. In seats with flexible backrests, ramping primarily manifested as an upward sliding of the torso, whereas in rigid structures, pelvis rebound and torso rotation around the *Y*-axis dominated. This change in the motion path affected the distribution of head accelerations and, as the video recordings suggest, may also have influenced bending moments in the lower cervical spine.

Another outcome of the study was the exponential relationship between HIC values and head acceleration and pulse parameters, confirmed by a high coefficient of determination (R^2^ > 0.95). This relationship, previously undescribed in the biomechanical literature for rear impacts, indicates the existence of a dynamic threshold beyond which the risk of head injury increases sharply. Most prior analyses [2,40] were based on neck injury criteria (NIC, Nkm), assuming a linear biomechanical response. In the present study, an alternative nonlinear model based on experimental data was proposed. This approach is supported by [19], which highlighted the lack of a clear correlation between Δv and clinical injury patterns and the nonlinearity of biomechanical responses in rear collisions. Such a consensus leads to the conclusion that new injury prediction models should consider not only the magnitude of accelerations but also their temporal structure and the trajectories of force transmission within the occupant’s body.

## 5. Conclusions

In this article, a series of sled tests were conducted using a Hybrid III dummy at four seatback recline angles and three crash pulse levels. The results were supplemented by an analysis of HIC values, acceleration durations, and response surfaces, enabling the identification of seat configurations associated with increased or decreased injury risk. Takin all inconsideration, the following can be concluded:

- There is a strongly nonlinear HIC–pulse relationship: The steepest local gradient was at 21°, whereas 25° lies in a more stable, slower-growth regime; even small geometric shifts materially alter occupant response.
- A new Pelvis-to-Headrest Transmission Effect was identified: Pelvis impact excites the seat structure, secondarily loading the headrest and producing a delayed head acceleration peak; this is especially relevant in semi-reclined postures and should inform restraint design.
- Greater recline generally reduced peak head acceleration and smoothed its growth: Higher angles triggered earlier *Z*-axis activity and extended the resultant acceleration duration. At 10 g, angle changes did not meaningfully alter the peaks (within SD).
- Increasing the crash pulse advanced the timing of head acceleration peaks relative to the crash pulse, highlighting the importance of timing in head–headrest interactions as collision energy rises.

## Figures and Tables

**Table 1:** Design of Experiments (DoE).

No	Crash Pulse	Seatback Recline Angle (SBA)	Measurement		
1	10 g	21°	Head resultant acceleration	Duration of the resultant head acceleration peak	Head Injury Criterion (HIC)
2	15 g	21°			
3	20 g	21°			
4	10 g	25°			
5	15 g	25°			
6	20 g	25°			
7	10 g	38°			
8	15 g	38°			
9	20 g	38°			
10	10 g	55°			
11	15 g	55°			
12	20 g	55°			

**Table 2:** Percentage increase in HIC values as a function of seatback recline angle and crash pulse magnitude.

SBA [°]	Increase in HIC: Crash Pulse 10 g → 15 g	Increase in HIC: Crash Pulse 15 g → 20 g
21	53.2%	83.3%
25	48.9%	50.7%
38	38.1%	77.6%
55	22.0%	78.0%

**Table 3:** Coefficients a, b, and R^2^ for each tested seatback recline angle.

SBA [°]	a	b	R^2^
21	14.64	0.10953	0.9934
25	19.90	0.08118	0.9999
38	14.49	0.09739	0.9826
55	15.09	0.08761	0.9505

## Data Availability

The raw data supporting the conclusions of this article will be made available by the author on request.

## Abbreviations

The following abbreviations are used in this manuscript:

ABTS	All-Belts-To-Seat
ASS-CDS/CISS	National Automotive Sampling System—Crashworthiness Data System/Crash Investigation Sampling System
ATD	Anthropomorphic Test Device
FMVSS	Federal Motor Vehicle Safety Standard
GHBMC	Global Human Body Models Consortium
HBM	Human Body Model
HIC	Head Injury Criterion
IIHS	Insurance Institute for Highway Safety
IIWPG	International Insurance Whiplash Prevention Group
IV-NIC	Inter-Vertebral Neck Injury Criterion
MSC	Motion Sequence Criteria
NDC	Neck Displacement Criterion
OOP	Out-of-Position
PMHS	Post-Mortem Human Surrogates
RCAR	Research Council for Automobile Repairs
SBA	Seatback Recline Angle
SIR	Seat-Integrated Restraint
THUMS	Total Human Model for Safety
WSTC	Wayne State Tolerance Curve
SD	Standard Deviation
